# The importance of education for understanding variability of dementia onset in the United States

**DOI:** 10.4054/demres.2024.50.26

**Published:** 2024-04-09

**Authors:** Hyungmin Cha, Mateo P. Farina, Chi-Tsun Chiu, Mark D. Hayward

**Affiliations:** 1 Shared authorship. Leonard Davis School of Gerontology, University of Southern California, Los Angeles, USA.; 2 Shared authorship. Department of Human Development and Family Sciences, Center on Aging and Population Sciences and Population Research Center, University of Texas at Austin, Austin, USA.; 3 Institute of European and American Studies, Academia Sinica, Taipei, Taiwan.; 4 Department of Sociology, Center on Aging and Population Sciences and Population Research Center, University of Texas at Austin, Austin, USA.

## Abstract

**BACKGROUND:**

Greater levels of education are associated with lower risk of dementia, but less is known about how education is also associated with the compression of dementia incidence.

**OBJECTIVE:**

We extend the literature on morbidity compression by evaluating whether increased levels of education are associated with greater dementia compression. We evaluate these patterns across race and gender groups.

**METHODS:**

We use the Health and Retirement Study (2000–2016), a nationally representative longitudinal study of older adults in the United States. To evaluate the onset and compression of dementia across education groups, we examine the age-specific distribution of dementia events, identifying the modal age of onset and the standard deviation above the mode (a measure of compression).

**RESULTS:**

While the modal age of onset is around 85 years among adults with a college degree, the modal age for adults with less than a high school education occurs before age 65 – at least a 20-year difference. The standard deviation of dementia onset is about three times greater for adults with less than a high school education compared to adults with a college degree. Patterns were consistent across race and gender groups.

**CONCLUSION:**

This research highlights the variability of dementia experiences in the older population by documenting differences in longevity without dementia and compression of dementia onset among more educated adults and less educated adults.

**CONTRIBUTION:**

We incorporate conceptual insights from the life span variability and compression literature to better understand education–dementia disparities in both the postponement and uncertainty of dementia onset in the US population.

## 1. Introduction

Dementia is and will continue to be a major population health concern in the United States. The number of people living with dementia is expected to double from 6 million in 2020 to 12 million in 2040 (Zissimopoulos et al. 2018). Prior research has shown a strong age patterning of dementia incidence, with the risk of dementia increasing twofold for every five years of chronological age (Castellani, Rolston, and Smith 2010; Hebert et al. 1995). However, the average age of onset for the population is still widely debated. A recent study estimated that the average age of onset was 83.7 years (Plassman et al. 2011), but other research suggests that the average age of onset may be shifting toward older ages due to declining mortality risk (Fishman 2017; Zissimopoulos, Crimmins, and St.Clair 2015).

Anticipating future trends is further complicated by the changing educational composition of the older adult population. Dementia risk at the individual and population level is strongly associated with educational attainment (Livingston et al. 2020; Lövdén et al. 2020; Zahodne and Zajacova 2020). Prior research has shown that declines in dementia prevalence are largely reflected in improvements in the education levels of older adults (Hayward et al. 2021). Other work has shown that higher levels of education are associated with longer lives, substantial lengthening of life without dementia, and a shorter period of life spent with dementia (Cha, Farina, and Hayward 2021; Crimmins et al. 2018; Farina et al. 2020; Hale et al. 2020; Sasson 2016).

We extend this line of work in several important ways. First, we assess whether later average ages of dementia onset associated with higher levels of education are also accompanied by a compression of dementia events. Compression refers to the length of lifetime spent living with dementia. Second, we assess how the cumulative rates of onset are associated with higher levels of education. Third, we evaluate how the basic patterns differ across gender and race groups. This descriptive work speaks to several common concerns in the population. Is dementia an inevitable consequence of growing older? What ages are the peak years for experiencing dementia onset? What can be done to significantly reduce the years that individuals live with dementia? A recent global survey documented that 80% of adults view dementia as inevitable, and 25% believe that there is nothing that can be done to prevent dementia (Alzheimer’s Disease International 2019). As our results show, that is far from the case.

Our approach draws on research examining life span variability in the mortality experiences of populations. This body of work is largely based on the question of whether improvements in mortality have also led to a compression of mortality into older ages or whether improvements have been accompanied by a shifting of the mortality distribution, where the shape of the distribution remains relatively unchanged (Canudas-Romo 2008; van Raalte, Sasson, and Martikainen 2018). Other research has begun to examine life span variability across social groups, such as educational and occupational groups, raising the question of whether life span variability is another marker of life span inequality across key groups (Aburto et al. 2020; van Raalte 2021; van Raalte, Martikainen, and Myrskylä 2014). For example, prior research has documented that higher levels of educational attainment are associated with not only longer life expectancy but also a significant compression of mortality. Well-educated individuals are able to maximize their life chances compared to less educated persons, resulting in a more compressed age distribution of when the onset of health conditions occurs (Brown et al. 2012; Edwards and Tuljapurkar 2005; van Raalte et al. 2011; Shkolnikov, Andreev, and Begun 2003).

Our study extends this line of inquiry to a major form of morbidity: dementia. As Stallard (2016) notes, patterns of life span variability and inequality for mortality need not be the same for morbidity. Thus educational differences in life expectancy could potentially result in compression or a shifting change in the distribution of morbidity events. To our knowledge, no research has examined this issue for the dementia experiences of an older population. Additionally, we investigate whether the educational differences in the average age of onset and compression vary across gender and race. Some recent evidence suggests that education is associated with significantly older ages of dementia onset for both older Black and older White Americans (Farina et al. 2020). However, it remains unclear whether the race groups experience similar levels of dementia compression. Differences in compression may point to other previously undetected forms of inequality present in the US population. Less attention has been given to gender, underscoring the need to document potential life span inequality for older men and women. The overarching goal of this study is to provide a richer demographic understanding of how educational inequality in dementia is experienced across important population subgroups. In particular, this study offers novel insights into educational differences in the compression of morbidity as it relates to dementia – a condition with broad ramifications for individuals, families, communities, and society as a whole.

## 2. Data and methods

### 2.1 Data

Our study is based on data from the 2000 to 2016 waves of the Health and Retirement Study (HRS). The HRS is a nationally representative, longitudinal study of US adults over the age of 50 (Sonnega et al. 2014). The HRS collects data approximately every two years and refreshes with new cohorts at younger ages every six years. Our analytic sample includes adults aged 65 years and older at each observation wave living in the community or a nursing home. This age restriction is due to the dementia classification scheme’s validation, which is not applicable to persons younger than 65 (Crimmins et al. 2011). Participants are allowed to enter the analytic sample when they reach the age of 65.

We initially started with 19,433 adults who were dementia-free in the first observation wave. We excluded respondents identifying as Hispanic and those classified under “Other” racial/ethnic categories (N = 2,330) due to statistical power issues, given their small numbers of dementia cases when stratified by education. Additionally, 900 adults who were not born in the United States were excluded due to concerns about the comparability of their education experiences. Another 215 adults were dropped because they had missing values on education. Our final analytical sample consists of 15,988 adults, of whom 3,581 experienced dementia onset. We constructed a person-years data file, suitable for estimating dementia incidence. Respondents contributed between 2 and 16 person-years of information, resulting in a total of 75,716 person-years of observation. Table 1 provides detailed information on the number of dementia cases and person-years, categorized by education, gender, and race.

### 2.2 Variables

#### Dementia Incidence.

HRS respondents’ cognitive status was classified using the Langa-Weir dementia approach, which relies on cognitive test scores for self-respondents and proxy scores for non-respondents. This classification scheme was validated by applying HRS cut points through a neuropsychological examination in the Aging Demographics and Memory Study, a subset of HRS respondents (Crimmins et al. 2011). Cognitive status assignment was based on either performance on several cognitive tests (self-respondents) or proxy information (proxy respondents). For self-respondents, scores ranged from 0 to 27 and comprised four components: immediate recall of ten words (scored from 0–10), delayed recall of the same ten words (0-10), five trials of Serial 7s (0–5), and backward counting (0–2). Respondents with scores ranging from 0 to 6 were classified as having dementia. Proxy scores ranged from 0–11 and were based on a proxy assessment of the respondent’s memory (0–4), limitations in instrumental activities of daily living (managing money, taking medication, preparing hot meals, using phones, and grocery shopping) (0–5), and the interviewer’s assessment of the respondent’s cognitive limitations during the interview (0–2). Respondents with scores ranging from 6 to 11 were classified as having dementia. To measure dementia incidence, we analyzed a change in cognitive status between waves from “no dementia” to “dementia.”

#### Mortality.

Since HRS participants could die before developing dementia, we included information about mortality as a competing risk in estimating the onset of dementia. Mortality incidence was based on the transition from a non-dementia status to death among persons at risk of dementia. The HRS tracks respondent mortality through two methods: the National Death Index (for mortality from 2000 to 2011) and exit interviews, during which proxy respondents provide information on deaths. The individual survival observations for HRS participants are considered accurate and representative (Weir 2016).

#### Covariates.

We categorized education into four groups based on the reported years of education: less than high school, high school, some college, and college. The age range of the participants was from 65 to 100. We determined participants’ exact age by calculating the number of years, months, and days between the date of birth and the interview date. Gender was indicated by a binary variable (1 for women, 0 for men), and race/ethnicity was self-reported (1 for non-Hispanic White and 0 for non-Hispanic Black).

### 2.3 Analytic plan

Our analysis consisted of three major steps. First, we used multivariate hazard models to estimate the transitions from no dementia to dementia and from no dementia to death. Second, we used cause-specific hazard models to calculate age-specific transition rates for dementia and mortality incidence. These rates allowed us to calculate multi-decrement life tables to simulate the life history dementia experience of a 100,000-person cohort. The life tables allow us to obtain the age-specific distribution of the number of dementia events (i.e., the decrement function due to dementia as a cause) in a standard population in the presence of mortality as a competing event. Third, based on the decrement function, we calculated the modal age of onset and the standard deviation above the mode to formally assess the variability in the distribution of dementia cases between ages 65 and 100. This approach is analogous to that used in a study of educational differences in mortality compression in the United States (Brown et al. 2012). We provide additional details below.

Dementia and mortality transition rates are defined by the equation below. Here $P_{ij}$ is the probability that a transition from state i (no dementia) to state j (either having dementia or dead) occurs in the age interval x to x+n, given that a person was in state i at age x.


$$\mu_{ij}(x)=\underset{n\to 0}{\text{lim}}\frac{P_{ij}(x,n)}{n}$$

Transition rates were estimated from multivariate hazard models using *Streg* in Stata 16.1. We assumed that rates within exposure intervals were constant. We also assumed that a dementia event occurred in the middle of an observation interval because cognitive status was observed only at the time of interview. We used information on the month and year of death to identify where, in an observation interval, deaths occurred. Additionally, attrition was treated as an unobserved censoring mechanism and was assumed to occur in the middle of an observation interval.

When estimating the cause-specific hazard models, we first stratified the analysis by gender and then by race. In the models stratified by gender, the predictors included age, race, and education. In the models stratified by race, gender was treated as a control variable. However, the density of events was too sparse to estimate hazard models for dementia risk within the race/gender subgroups. The parameter estimates of the covariates allowed us to calculate education-specific predicted risks of dementia/mortality at each age for men and women while controlling for race. Similarly, the parameter estimates obtained when stratifying by race allowed us to calculate education-specific predicted risks of dementia/mortality at each age for Black adults and White adults while controlling for gender.

The statistical models applied the baseline year weight to account for the complexity of the HRS sampling designs (HRS 2019). However, for the race-stratified models, we did not use sampling weights. The HRS sampling weights are designed to adjust for oversampling among minority populations and non-response. Despite being intended to represent the national population, they may not ensure national representativeness for subpopulations for Black and White adults (Gouskova 2013). As a sensitivity check, we used the sampling weights, but they did not alter the main findings.

The age-specific schedules of dementia onset and mortality were then used to calculate multi-decrement life tables for the education groups, which were stratified by gender (eight life tables) and by race (eight life tables) (Hayward, Hardy, and Grady 1989; Laditka and Hayward 2002; Land, Guralnik, and Blazer 1994; Teachman and Hayward 1993). Each life table simulates the lifetime dementia experiences of a 100,000-person cohort defined by the covariates. (Table A-1 provides an example of a multi-decrement life table.)

For each life table, we adopted an approach based on Kannisto (2001)’s work. This approach allowed us to explicitly evaluate both longevity and variability in the distribution of lifetimes without dementia. We assessed longevity using the modal age of experiencing dementia, while the standard deviation above the mode captures the degree of variability or compression. These metrics enabled us to assess how education was associated with the distribution of experiencing dementia across population subgroups.

Since the metric measures the upward standard deviation, the standard deviation above the mode is less likely to be influenced by bias due to a fixed age limit (Kannisto 2000, 2001). Since our age range starts at 65, the standard deviation above the mode serves as an appropriate metric for measuring variability. We calculated the standard deviation above the mode by summing the squared positive deviations from the modal age of dementia and dividing this sum by the number of age intervals above the mode. We assumed that dementia cases were uniformly distributed within a specific age interval (Cheung et al. 2005). Smaller values for the standard deviation above the mode indicate greater levels of dementia compression.

Additionally, we used the multi-decrement life table decrement function to calculate the lifetime risk of dementia. Lifetime risk represents the proportion of adults in each life table cohort who will eventually experience dementia based on age-specific dementia and mortality incidence rates in the HRS from 2000 to 2016 for those who survived to age 65 (Preston, Heuveline, and Guillot 2000). The lifetime risk was based on the summation of the age-specific number of dementia events between ages 65 and 100 and dividing the result by the radix (i.e., 100,000). We estimated age-specific cumulative risks (e.g., by age 70, 80, and 90) and the lifetime risk of dementia onset for those without dementia at age 65.

## 3. Results

### 3.1 Sample characteristics

Table 2 presents descriptive statistics for an unweighted baseline analytical sample drawn from the HRS conducted between 2000 and 2016, with a total sample size of 15,988. The table includes the sample’s mean age, which is 71.18 years, with a standard deviation of 7.13. Approximately 57% of the sample consists of women. One-quarter of the sample did not graduate from high school, while 35% completed high school, 20% had some college education, and another 20% had achieved a college degree or higher. Additionally, approximately 16% of the sample identified as Black.

#### 3.1.1 Age-specific incidence rate

**Figure 1:**
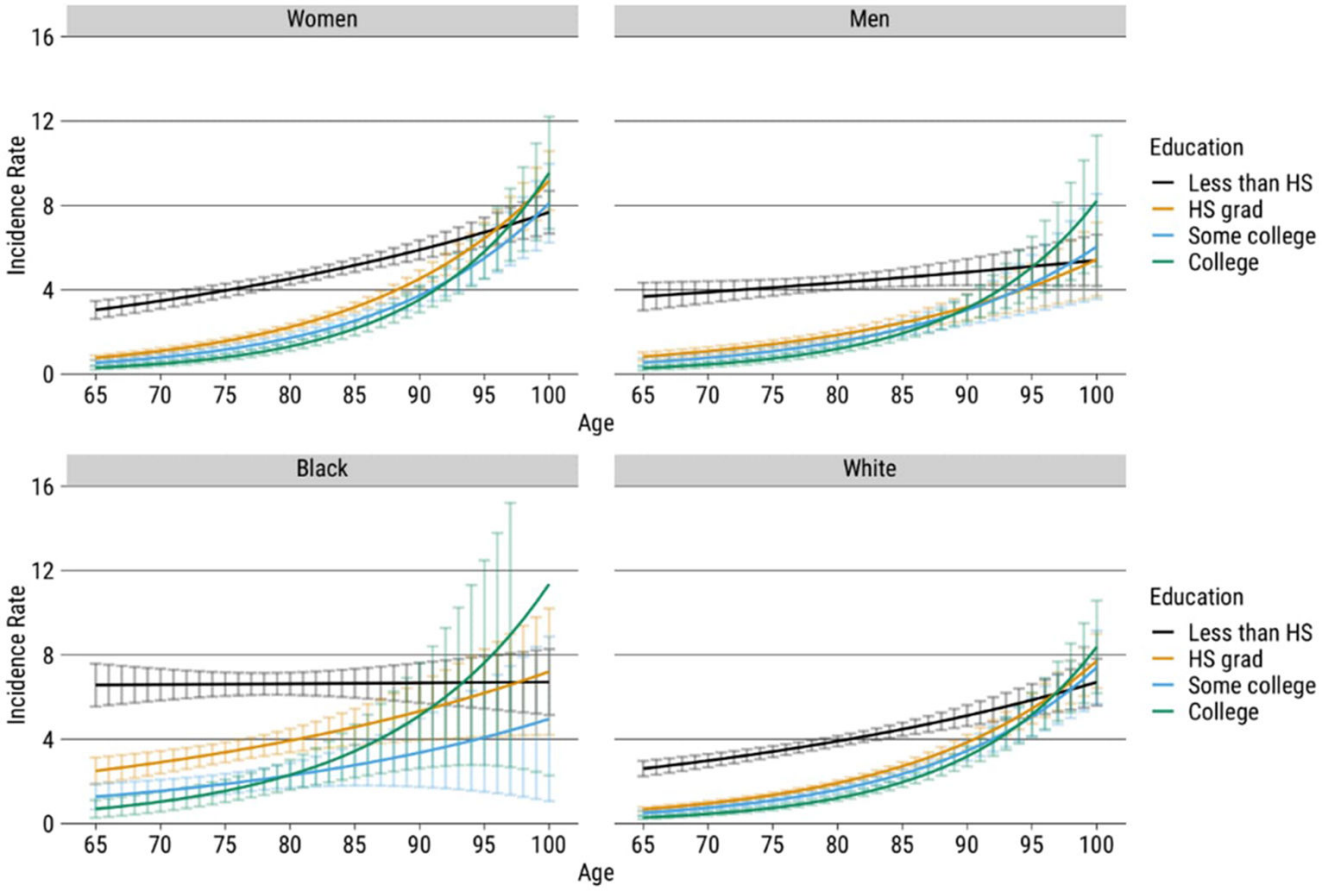
Age-specific incidence rate by education, gender, and race, HRS 2000–2016 *Note*: The *y*-axis represents the incidence of dementia per 100 cases. Confidence intervals represent a 95% level of confidence.

Before addressing our main research aims, Figure 1 presents the predicted incidence rates of dementia from our hazard models across various age groups. These age groups are categorized by education, gender, and race. Detailed results of the hazard models can be found in Tables A-2 and A-3.

As age increases from 65 to 100, the incidence rate shows an upward trend across all categories. The figures show that different educational levels exhibit distinct age-specific patterns. Specifically, individuals who did not graduate from high school have significantly higher risks of dementia at younger ages, and this trend persists for all groups until approximately age 85–90, depending on the group. In contrast, patterns of age-specific incidence rates for high school graduates, individuals with some college education, and college graduates are generally similar to each other. College graduates exhibit the lowest risks of dementia until advanced ages, when they begin to rise rapidly.

#### 3.1.2 Modal age of onset and standard deviation above the mode

**Figure 2:**
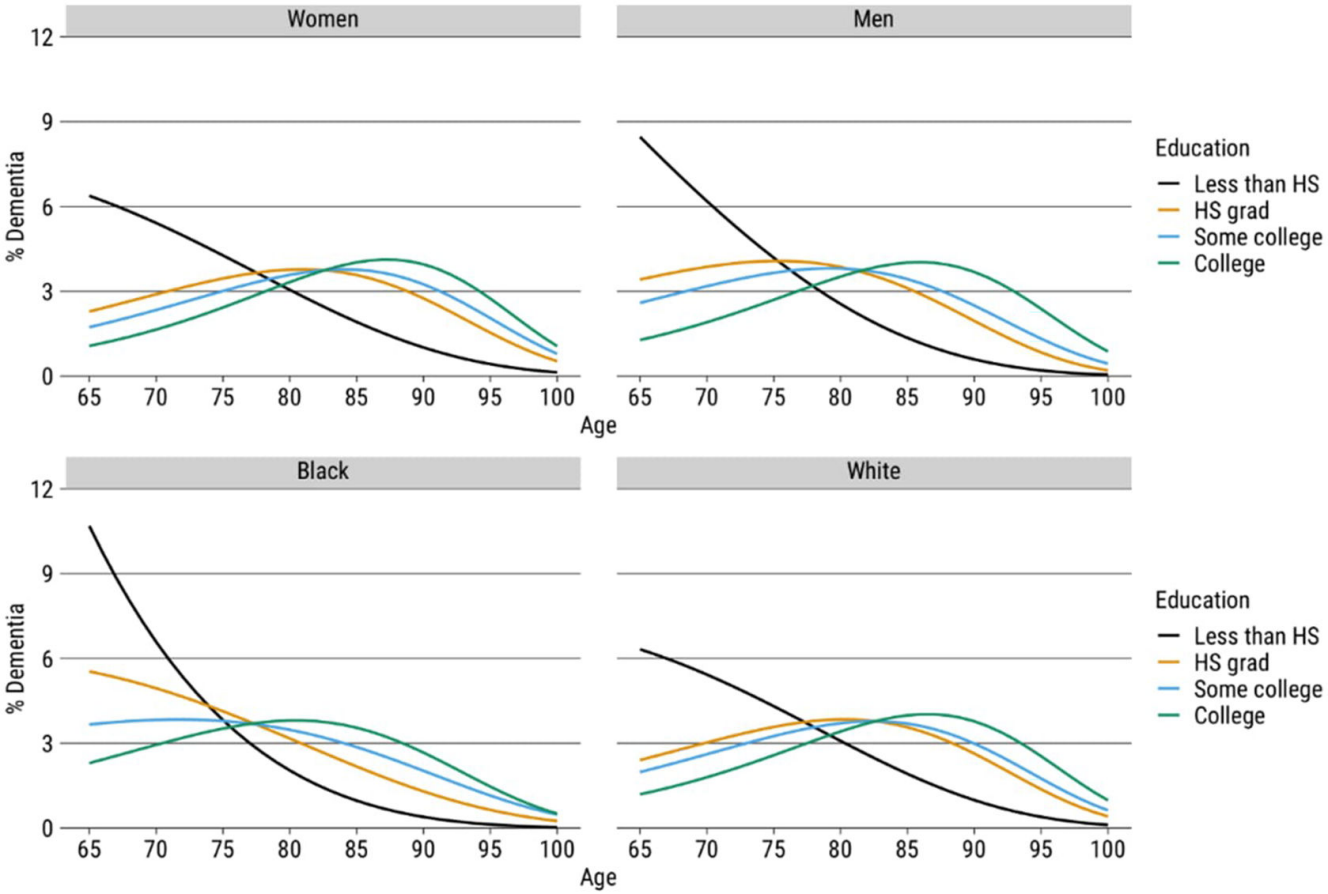
Age-specific percentage of life table dementia cases by education, gender, and race, HRS 2000–2016 *Note*: The *y*-axis represents the percentage of dementia per 100 cases. The numerator represents the count of dementia cases at each age, while the denominator corresponds to the total number of dementia cases between ages 65 and 100, which we then multiplied by 100.

The findings presented in Figure 2 and Table 3 show the association between education and both the distribution of ages at which dementia occurs and the variability of that distribution. Figure 2 shows that higher levels of education are associated with a delayed onset of dementia to older ages, as evidenced by an increase in the modal age of dementia onset with higher education levels. Note, however, that those without a high school education already reach the peak age of dementia distribution among persons who are at risk of dementia at age 65. This points to an accelerated dementia process among persons with the lowest level of educational attainment.

Table 3 summarizes the graphs, showing the modal age of dementia onset and the standard deviation above the mode by levels of education by gender and race. The results in panels A and B show that both women and men have educational gradients in modal age of dementia onset as well as increased compression at higher levels of education. For example, the gap in women’s modal age between high school and college is 6.31 years (81.56 years compared to 87.88). The gap in men’s modal age is 10.46 years (75.99 years compared to 86.45). The women’s level of compression drops from 11.49 years to 7.75 years between high school and college, and the men’s level drops from 14.59 years to 8.7 years. In addition, the results also highlight that the levels of dementia compression are higher for women at all levels of education.

Panels C and D present parallel results for Black and White adults. Higher levels of education are associated with a delayed onset of dementia for both Black and White adults. Black adults who have less than a high school diploma have a comparable modal age of about 66 years. This reflects both the high levels of risk for these groups as well as a “radix effect,” which represents the persons in the life table population at age 65 at risk of experiencing dementia. The modal age gap for Black adults with a high school and college education is substantial, with a difference of 15 years (65.98 years compared to 81.31 years). In contrast, the modal age for White adults with a college education is 87.09, while for White adults with a high school education, it is 80.97, showing an approximate six-year difference.

While both Black adults and White adults benefit in terms of later ages of dementia with higher levels of education, Black adults lag far behind White adults in their longevity without dementia. A parallel pattern is evident for compression. While both Black adults and White adults show significant increases in compression with higher levels of education, compression levels are much lower among Black adults. For example, the level of compression for Black adults with a college education is 11.71 years, the same as for White adults who have only a high school education.

### 3.2 Cumulative and lifetime risk of dementia

Not surprisingly given the results above, the lifetime risk of experiencing dementia shown in Table 4 differs substantially across education levels. Persons with less than a high school education are dramatically more likely to experience dementia in their lifetime compared to all other education groups. For example, among women, by age 70, 15.99% of those with less than a high school education are expected to develop dementia, compared to only 2.16% of college-educated women. This gap persists at age 90 (43.40% versus 19.62%), with the lifetime risk of dementia onset being approximately 20% higher for women with less than a high school education compared to women with a graduate degree. Similar patterns are observed among men. For instance, 41.83% of men with less than a high school education are expected to experience dementia by age 100, whereas only 21.62% of college-educated men are expected to face dementia risk by age 100.

While Black adults also exhibit educational gradients in lifetime cumulative risk, their overall risk of developing dementia is higher than that of White adults. By age 70, 30.10% of Black adults with less than a high school diploma are expected to experience dementia onset, compared to 14.29% of White adults with the same education level. By age 90, among Black adults without a high school diploma, 58.08% face a lifetime risk of dementia, while 38.81% of White adults with less than a high school diploma are expected to develop dementia by age 90. While there is a clear educational gradient among Black adults in terms of lifetime risk of dementia within the population, it is noteworthy that the lifetime risk of even college-educated Black adults is similar to that of White adults with only a high school diploma.

Overall, the lifetime risk of developing dementia ranges from 40.50% to 58.92% for those with less than a high school diploma, with Black adults having the highest risk within this group. Moreover, higher levels of education are associated with a lower lifetime risk of dementia. For women, graduating from college is associated with an 18.91% difference in lifetime risk of dementia, reducing it from 45.43% to 26.52% compared to those who did not graduate from high school. Similarly, for men, graduating from college leads to a 20.21% difference in lifetime risk of dementia, decreasing it from 41.83% to 21.62% compared to those who did not graduate from high school. Among Black adults, obtaining a college degree is associated with a 29.14% difference in the lifetime risk of dementia, reducing it from 58.92% to 29.78% compared to those with less than a high school education. Conversely, among White adults, obtaining a college degree is associated with a 17.14% reduction in the lifetime risk of dementia, lowering it from 40.50% to 23.36%. However, across all population subgroups, except for Black adults, the most substantial decline in dementia risk occurs once adults graduate from high school.

## 4. Discussion

While the link between educational attainment and dementia risk is well documented (see Lövdén et al. 2020 for a review), much less is known about the inequality of dementia risk *within* levels of education. Population health studies have suggested that higher education confers a coalescence of resources and a minimization of risks that leads to a compression of disability, morbidity, or mortality at older ages (Brown et al. 2012; Cai and Lubitz 2007; Chiu et al. 2018; Leigh and Fries 1994; van Raalte et al. 2011; van Raalte, Sasson, and Martikainen 2018). To the extent that this pattern is mirrored in dementia risk, it has implications for understanding the factors that contribute to both similarities and differences in lifetime dementia experiences among individuals with varying levels of education. This, in turn, can enhance public health awareness and responses to dementia risk in the United States. As evident from the analysis, we find strong evidence indicating that higher levels of educational attainment not only delay the onset of dementia but also result in a compression of dementia into later stages of life. This evidence further supports the notion that individuals with higher levels of education maximize their lifetime opportunities and minimize risks, ultimately reducing the variability in the timing of dementia among the well-educated population.

In this study, we draw on the concept of the modal age to gauge educational differences in the average life span without dementia. As is evident, there are stark differences in the modal age of dementia between the most and least educated adults, varying by approximately 16 to 23 years. This has substantial implications for the health of people who differ by education. The earlier onset of dementia for the least educated indicates that cognitive health deterioration is occurring decades earlier in life, which can translate to worse quality of life overall, especially considering the substantial impacts that cognitive impairment can have on activities of daily living and maintaining one’s independence (Giebel, Sutcliffe, and Challis 2015; Nakayama et al. 2017). Educational differences in modal ages of dementia surpass those observed for other health outcomes (Brown et al. 2012; Chiu et al. 2018), pointing to the outsized importance of education in shaping dementia risk.

In addition, it is important to note that the actual difference between modal ages of the least and most educated groups in the population may be greater than what is observed in this study. Due to data limitations, we were unable to obtain dementia status classification for adults prior to age 65. For adults with a high school diploma or greater, this data limitation has a minimal impact on understanding the underlying form of dementia incidence risk because dementia incidence at 65 remains low and then gradually increases in later ages. In contrast, we are uncertain about the shape of the incidence curve for adults with less than a high school diploma given that in our models, dementia incidence rates decline after age 65, which may be in part related to the high initial rates occurring prior to 65 that we cannot observe. More research is needed to better understand the compression of dementia onset for people with less than a high school education. Therefore inequalities observed in this study should be considered to be more conservative than inequalities in the actual population.

Our analysis also makes clear that the dementia experiences of well-educated people are compressed, and the compression occurs at very advanced ages compared to the least educated adults. This is evident through differences in the modal age of onset and the standard deviation above the mode across groups. Prior studies have found similar patterns for mortality and disability (Brown et al. 2012; Chiu et al. 2018; van Raalte, Sasson, and Martikainen 2018). Studies that have documented dementia life expectancies have also found significant compression (few years lived with dementia) with increased educational attainment (Cha, Farina, and Hayward 2021; Farina et al. 2020; Garcia et al. 2021). Overall, compression of dementia indicates that well-educated adults appear to be reaping the maximum benefits of their advantaged status to minimize their risk and to put off dementia onset until advanced ages.

While population health research has often focused on the numerous advantages of college-educated groups as an explanation for compression, less attention has been given to understanding the high level of variation in dementia experience among the less educated. While prior research on life span variability in mortality has characterized high levels of variation in terms of greater uncertainty (van Raalte 2021; van Raalte, Sasson, and Martikainen 2018), less attention has been given to underlying causes of greater uncertainty among less educated adults. One promising area of research that may provide insight into differences in variation of morbidity and mortality across education groups is the role of state policies (Brown, Lariscy, and Walker 2023; Montez 2017; Montez et al. 2019, 2020). In the mortality literature, the mortality profiles of college-educated adults appear similar across states, regardless of their policy environments. In contrast, adults with fewer years of education vary widely across states due to differences in economic and social opportunities, as well as health behaviors affected by the overall policy environment. As a result, it is believed that individuals with lower levels of education are more affected by state policies, which may contribute to greater variability in health outcomes. Understanding how the overall context and individual opportunities impact health across education groups may provide additional insights into why more uncertainty exists among people with less education and should be extended to other forms of health inequalities, such as dementia.

The literature on the association between education and health also makes it clear that the patterns reported here are likely to be dynamic and will change over time. While this study cannot directly examine these changes, we suggest that future research attend to historical changes in the economic and social opportunities for adults without a high school diploma – and even adults with a college education – that occur over time. For example, a significant number of older adults without a high school diploma analyzed in this study may have found stable employment, better housing, and other social and economic opportunities, since the youngest in this sample would have reached adulthood in the late 1960s, which preceded the decline of blue-collar work and the increase in downward mobility (or greater stagnation) in the United States in the following decades (Acs 2011; Chetty et al. 2017; Hout 2018). Future research might show that dementia incidence is more compressed for adults with lower levels of education too, as social and economic dynamics that limit opportunities and exposures have changed over the last few decades, thereby narrowing the channels of dementia risk.

While the later onset and greater compression of dementia with increased educational attainment was found across gender and race groups, notable differences between groups were also evident. As found in other studies, Black older adults experienced dementia at earlier ages than White older adults across all education groups (Derby et al. 2017; Farina et al. 2020; Powell et al. 2021). This inequality was also reflected in differences found for cumulative onset, where Black older adults have a rate that is roughly twice that of White older adults across all age and education groups. This further illustrates the importance of considering how race-based inequalities are propagated even with increased educational attainment that confers social and economic opportunities. Gender differences were most evident for those with high school and some college education, with men experiencing earlier onset and greater variability than women. Greater parity was observed with increased levels of education. Men’s earlier onset and greater variability may be partly tied to health behaviors, as men are more likely to engage in behaviors that are detrimental to health. For example, men have greater levels of smoking (Bauer, Göhlmann, and Sinning 2007), and smoking has been tied to increased dementia risk (Peters et al. 2008; Zhong et al. 2015). However, as education increases, health behaviors improve and gender differences subside, as is the case with smoking (Pampel et al. 2017), which may narrow the differences in morbidity compression for conditions like dementia. This may be one of many pathways that contribute to the earlier onset and greater variability of dementia among men. Future research should further consider how gendered experiences may be associated with cognitive health risk later in life. Lastly, it is important to note that this data cannot provide insight into gender and race differences for the less than high school group, as left censoring at age 65 prevents a meaningful evaluation.

## 5. Limitations

While this study provides many advancements to understanding educational inequality in dementia risk, important limitations should be acknowledged. The Langa-Weir classification is an imperfect measure (Crimmins et al. 2011) and is not based on doctor diagnosis, which may lead to some measurement error. This measurement error cannot be fully accounted for in these models. However, it is important to note that it is one of the most widely used population-based measurements of dementia classification, which allows for some indirect comparison of our findings with other studies, and it is one of the best-performing algorithms when evaluated against Medicare claims when estimating population prevalence (Chen et al. 2019). Therefore we do not believe that our results and the conclusions drawn from them will be greatly impacted by the choice of measurement. However, it is also important to note that socially disadvantaged groups may experience dementia earlier than other groups (as is the case with less educated Black older adults in this study), meaning that we are unable to fully evaluate the population dynamics of dementia for some of the most disadvantaged groups. Future research may consider validating and collecting cognitive status information for socially disadvantaged groups at earlier ages to better understand this phenomenon. Additionally, we exclude Hispanics, Asians, and respondents classified as “Other” due to sample size concerns. Future work using datasets that provide large and robust samples on these groups should evaluate whether the same patterns observed in this study hold for these other groups, which will make up an increasingly larger share of the older adult population. Lastly, our modeling approach does not allow for the direct examination of statistical differences across subgroups (Laditka and Hayward 2002). We can evaluate differences in patterns only within groups, not across them. With more data, future research should consider using a bootstrapping microsimulation approach to provide further insight into subgroup differences by estimating statistical properties of the underlying distribution of dementia events (Cai et al. 2010).

## 6. Conclusions

Our research indicates that dementia onset, similar to the variability in senescence (Jones and Vaupel 2017; Vaupel 2010), is amenable to educational attainment. Specifically, our findings demonstrate that higher levels of education are associated with a delayed onset of dementia and a greater degree of compression. To our knowledge, no prior studies have evaluated these differences. These findings have broad implications for understanding the future of US population health and educational health inequalities. Dementia prevalence has been decreasing in the US population (Chen and Zissimopoulos 2018; Farina et al. 2022; Hayward et al. 2021). Much of the decrease has been attributable to improvements in educational attainment (Hayward et al. 2021; Langa et al. 2017). This study shows that part of the underlying process in improvements may be in delayed onset and greater compression. To the extent that gains in educational attainment continue, the modal age of dementia incidence for the overall population may be pushed further into older age, improving health for US adults in their 70s and 80s and lowering dementia prevalence. However, recent evidence points to a stalling in the upward US educational trend, which also is likely to be reflected in future peak ages of onset and compression (Heckman and LaFontaine 2010). Regarding inequality, the greatest differences were observed for the less than high school and high school groups. However, differences among adults with high school and greater levels of education may grow in the future. Recent health metrics have shown a deterioration of health for those without a college degree, including earlier death and increased variability in timing of death (Montez and Zajacova 2013, 2014; Sasson 2016; Sasson and Hayward 2019). If dementia experiences follow a similar change, then inequalities may grow in the coming years.

## Figures and Tables

**Table 1:** Number of dementia and death events and person-years of exposure to the risk of dementia and death in the HRS by education, gender, and race

	Dementia		Death	
	Event	Exposure	Event	Exposure
Women (N = 9,038)				
Less than high school	937	10,137	1,323	10,137
High school	777	17,798	1,546	17,798
Some college	310	9,072	689	9,072
College	181	6,738	448	6,738
Men (N = 6,950)				
Less than high school	684	7,856	1,175	7,856
High school	344	10,146	1,073	10,146
Some college	167	5,819	604	5,819
College	181	8,150	670	8,150
Black adults (N = 2,476)				
Less than high school	634	4,944	679	4,944
High school	212	3,050	273	3,050
Some college	66	1,685	116	1,685
College	41	1,135	94	1,135
White adults (N = 13,512)				
Less than high school	987	13,049	1,819	13,049
High school	909	24,894	2,346	24,894
Some college	411	13,206	1,177	13,206
College	321	13,753	1,024	13,753

*Note*: A total of 15,988 adults contributed 75,716 person-years.

**Table 2:** Unweighted descriptive statistics of analytic sample at baseline, HRS 2000–2016 (N = 15,988)

	Mean/Prop.	SD
Age	71.18	7.13
Women	0.57	
Education		
Less than high school	0.25	
High school	0.35	
Some college	0.20	
College or more	0.20	
Black	0.16	

**Table 3:** Modal age of dementia onset and standard deviation above the mode by education, gender, and race, HRS 2000–2016

	Modal age	SD(M+)
*Panel A: Women*		
Less than high school	65.97	20.37
High school	81.57	11.49
Some college	84.63	9.70
College	87.88	7.75
*Panel B: Men*		
Less than high school	65.95	20.40
High school	75.99	14.59
Some college	79.80	12.44
College	86.45	8.70
*Panel C: Black adults*		
Less than high school	65.92	20.42
High school	65.98	20.37
Some college	72.27	16.94
College	81.31	11.71
*Panel D: White adults*		
Less than high school	65.98	20.37
High school	80.97	11.71
Some college	83.24	10.61
College	87.09	8.44

**Table 4:** Cumulative onset of dementia at age *x* by education for gender and race groups, HRS adults aged 65 and older (2000–2016)

	Age *x*			
	Age 70	Age 80	Age 90	Lifetime risk
*Panel A: Women*				
Less than high school	15.99	34.83	43.40	45.43
High school	5.09	16.46	27.78	32.67
Some college	3.69	12.99	24.05	29.92
College	2.16	8.98	19.62	26.52
*Panel B: Men*				
Less than high school	18.22	35.27	40.91	41.83
High school	5.08	14.40	21.32	23.31
Some college	3.58	11.12	17.93	20.59
College	2.07	8.18	16.74	21.62
*Panel C: Black adults*				
Less than high school	30.10	52.30	58.08	58.92
High school	13.96	31.82	41.26	44.15
Some college	7.76	20.60	30.11	33.95
College	4.63	15.06	25.34	29.78
*Panel D: White adults*				
Less than high school	14.29	31.22	38.81	40.50
High school	4.56	14.59	24.21	28.02
Some college	3.53	11.98	21.26	25.73
College	2.11	8.46	17.71	23.36

*Note*: The lifetime risk of developing dementia is the chance that an individual who is dementia-free at age 65 will develop dementia by the time they reach age 100.

## Appendix

**Table A-1:** A sample multi-decrement life table for women with less than a high school education

Age	Dementia	Death	m(x)	l(x)	L(x)	d(dem)	d(death)	l(dem)	l(death)
65	0.03	0.02	0.05	100000.00	97476.08	2867.13	2181.85	45605.57	54488.96
66	0.03	0.02	0.05	94952.15	92451.55	2792.14	2210.29	42738.44	52307.11
67	0.03	0.03	0.06	89950.95	87478.38	2712.66	2233.80	39946.30	50096.82
68	0.03	0.03	0.06	85005.81	82566.12	2628.87	2251.92	37233.64	47863.03
69	0.03	0.03	0.06	80126.44	77724.61	2540.97	2264.22	34604.77	45611.11
70	0.03	0.03	0.06	75322.78	72963.88	2449.19	2270.26	32063.80	43346.89
71	0.03	0.03	0.07	70604.97	68294.13	2353.80	2269.65	29614.61	41076.63
72	0.04	0.04	0.07	65983.28	63725.64	2255.14	2262.04	27260.81	38806.98
73	0.04	0.04	0.07	61467.99	59268.68	2153.57	2247.08	25005.66	36544.94
74	0.04	0.04	0.08	57069.36	54933.43	2049.48	2224.53	22852.09	34297.86
75	0.04	0.04	0.08	52797.50	50729.90	1943.32	2194.19	20802.62	32073.32
76	0.04	0.05	0.09	48662.29	46667.75	1835.56	2155.94	18859.30	29879.13
77	0.04	0.05	0.09	44673.22	42756.27	1726.73	2109.73	17023.74	27723.19
78	0.04	0.05	0.09	40839.33	39004.18	1617.37	2055.64	15297.00	25613.46
79	0.04	0.06	0.10	37169.03	35419.52	1508.05	1993.82	13679.63	23557.82
80	0.04	0.06	0.10	33670.01	32009.54	1399.34	1924.56	12171.59	21564.00
81	0.04	0.06	0.11	30349.08	28780.58	1291.87	1848.25	10772.24	19639.43
82	0.05	0.07	0.11	27212.08	25737.88	1186.22	1765.40	9480.38	17791.18
83	0.05	0.07	0.12	24263.69	22885.55	1082.99	1676.64	8294.16	16025.78
84	0.05	0.08	0.13	21507.40	20226.37	982.78	1582.72	7211.17	14349.14
85	0.05	0.08	0.13	18945.33	17761.77	886.13	1484.51	6228.40	12766.42
86	0.05	0.09	0.14	16578.21	15491.74	793.57	1382.94	5342.27	11281.91
87	0.05	0.10	0.15	14405.28	13414.76	705.57	1279.07	4548.70	9898.97
88	0.05	0.10	0.16	12424.24	11527.78	622.55	1174.00	3843.13	8619.89
89	0.06	0.11	0.16	10631.32	9826.27	544.87	1068.85	3220.58	7445.90
90	0.06	0.12	0.17	9021.22	8304.21	472.80	964.80	2675.72	6377.04
91	0.06	0.12	0.18	7587.21	6954.21	406.53	862.97	2202.92	5412.24
92	0.06	0.13	0.19	6321.22	5767.61	346.19	764.45	1796.39	4549.27
93	0.06	0.14	0.20	5213.99	4734.61	291.79	670.27	1450.20	3784.82
94	0.06	0.15	0.21	4255.23	3844.50	243.28	581.32	1158.40	3114.55
95	0.06	0.16	0.23	3433.78	3085.83	200.50	498.37	915.12	2533.24
96	0.07	0.17	0.24	2737.88	2446.63	163.22	422.04	714.62	2034.87
97	0.07	0.18	0.25	2155.39	1914.70	131.16	352.78	551.40	1612.82
98	0.07	0.20	0.27	1674.02	1477.80	103.94	290.82	420.24	1260.05
99	0.07	0.21	0.28	1281.59	1123.93	81.17	236.24	316.30	969.23
100	0.07	0.22	0.30	966.28	841.54	62.40	188.93	235.14	732.99

**Table A-2:** Coefficients from accelerated failure time models by gender and education

	Dementia incidence	Mortality incidence
Women		
Less than high school (N = 10,137 person-years)		
Age	−0.03*** (0.00)	−0.07*** (0.00)
Black	−0.61*** (0.06)	−0.08 (0.06)
Constant	5.37*** (0.30)	8.10*** (0.28)
High school (N = 17,798 person years)		
Age	−0.07*** (0.00)	−0.09*** (0.00)
Black	−0.72*** (0.09)	−0.04 (0.09)
Constant	9.54*** (0.35)	10.37*** (0.29)
Some college (N = 9,072 person-years)		
Age	−0.08*** (0.01)	−0.10*** (0.00)
Black	−0.50** (0.18)	−0.15 (0.14)
Constant	10.33*** (0.56)	11.15*** (0.41)
College (N = 6,738 person-years)		
Age	−0.10*** (0.01)	−0.11*** (0.01)
Black	−0.76*** (0.21)	−0.36* (0.16)
Constant	12.36*** (0.63)	11.93*** (0.51)
Men		
Less than high school (N = 7,856 person-years)		
Age	−0.01* (0.01)	−0.06*** (0.00)
Black	−0.47*** (0.08)	−0.04 (0.08)
Constant	4.12*** (0.44)	7.73*** (0.35)
High school (N = 10,146 person years)		
Age	−0.05*** (0.01)	−0.09*** (0.01)
Black	−0.93*** (0.15)	−0.10 (0.16)
Constant	8.39*** (0.59)	10.06*** (0.43)
Some college (N = 5,819 person-years)		
Age	−0.07*** (0.01)	−0.08*** (0.01)
Black	−0.42 (0.23)	0.28 (0.18)
Constant	9.73*** (0.88)	9.33*** (0.46)
Men		
College (N = 8,150 person-years)		
Age	−0.10*** (0.01)	−0.10*** (0.01)
Black	0.04 (0.39)	−0.64** (0.20)
Constant	12.14*** (0.89)	10.99*** (0.42)

*Note*: *** p < .001, ** p < .01, * p < .05. Standard errors are in parentheses.

**Table A-3:** Coefficients from accelerated failure time models by race and education

	Dementia Incidence	Mortality Incidence
Black adults		
Less than high school (N = 4,944 person-years)		
Age	−0.00 (0.01)	−0.06*** (0.00)
Female	−0.11 (0.08)	0.22** (0.08)
Constant	2.83*** (0.40)	7.67*** (0.35)
High school (N = 3,050 person-years)		
Age	−0.03*** (0.01)	−0.06*** (0.01)
Female	−0.04 (0.15)	0.36** (0.12)
Constant	5.67*** (0.68)	7.46*** (0.53)
Some college (N = 1,685 person-years)		
Age	−0.04* (0.02)	−0.08*** (0.01)
Female	0.02 (0.25)	0.19 (0.19)
Constant	6.86*** (1.32)	9.37*** (0.94)
College (N = 1,135 person-years)		
Age	−0.08*** (0.02)	−0.07*** (0.01)
Female	−0.51 (0.40)	0.51* (0.21)
Constant	10.51*** (1.46)	8.53*** (1.01)
White adults		
Less than high school (N = 13,049 person-years)		
Age	−0.03*** (0.00)	−0.07*** (0.00)
Female	0.05 (0.06)	0.26*** (0.05)
Constant	5.37*** (0.33)	7.91*** (0.24)
White adults		
High school (N = 24,894 person-years)		
Age	−0.07*** (0.00)	−0.09*** (0.00)
Female	−0.17* (0.07)	0.35*** (0.04)
Constant	9.61*** (0.35)	10.22*** (0.22)
Some college (N = 13,206 person-years)		
Age	−0.08*** (0.01)	−0.09*** (0.00)
Female	−0.08 (0.10)	0.48*** (0.06)
Constant	10.29*** (0.50)	10.04*** (0.30)
College (N = 13,753 person-years)		
Age	−0.10*** (0.01)	−0.10*** (0.00)
Female	−0.06 (0.11)	0.26*** (0.06)
Constant	12.16*** (0.58)	11.13*** (0.33)

*Note*: *** p < .001, ** p < .01, * p < .05. Standard errors are in parentheses.
